# Reaction to ‘addressing key limitations in the study on DCIS response to neoadjuvant chemotherapy in TNBC’

**DOI:** 10.1016/j.breast.2025.104467

**Published:** 2025-04-04

**Authors:** Eva L. Claassens, Roxanne A.W. Ploumen, Thiemo J.A. van Nijnatten, Marjolein L. Smidt

**Affiliations:** a Department of Surgery, Maastricht University Medical Center+, Maastricht, the Netherlands; b GROW – Research Institute for Oncology and Reproduction, Maastricht University, Maastricht, the Netherlands; c Department of Radiology and Nuclear Medicine, Maastricht University Medical Center+, Maastricht, the Netherlands

We appreciate the perceptive commentary provided by dr. Venkataraman and prof. Mokbel on our manuscript ‘The effect of neoadjuvant chemotherapy on ductal carcinoma in situ in triple-negative breast cancer patients: A nationwide analysis’ [1]. Their insights and concerns contribute to the ongoing discussion on DCIS response to systemic therapy, and we welcome the opportunity to clarify and further contextualize our findings.

Firstly, the authors express concern regarding the reported DCIS pCR rate of 53.6 %, and the potential confounding effect of biopsy sampling, which may result in underdetection of DCIS. We acknowledge that pre-neoadjuvant chemotherapy (NACT) biopsies may miss the DCIS component when present, due to sampling limitations. We explicitly addressed this in the discussion section of our manuscript, recognizing the risk of underdetection of DCIS and the possible impact on the observed response rate. However, this challenge is not unique to our study but is a recognized limitation of all retrospective analyses relying on pre-NACT histopathology. Furthermore, our results align with existing literature, including the authors’ own pooled analysis, reinforcing the reproducibility of our findings [[2], [3], [4]]. To ensure the most accurate assessment of response rates, we only included patients in whom the presence of DICS was confirmed pre-NACT. Consequently, there are likely additional patients with undetected DCIS in our cohort for whom response assessment was not possible. In theory, this could have led to either an overestimation or underestimation of the true DCIS pCR rate, but the direction of this potential bias remains uncertain. While this introduces some uncertainty, our observed pCR rate demonstrates the possibility of DCIS pCR in more than half of TNBC patients, challenging the assumption that DCIS is irresponsive to NACT.

Secondly, the authors point out that in our study, the absence of calcifications may predict a greater likelihood of DCIS response, however this was excluded from our multivariable analysis due to missing data. Additionally, they indicate that the lack of imaging-pathology correlation is a limitation, a point with which we agree. As noted by the authors and in our discussion section, persistent calcifications on imaging do not necessarily correlate with residual DCIS. However, our study was not designed to evaluate the imaging-pathology correlation. Instead, we aimed to highlight the potential predictive role of calcifications present in the biopsy. Currently, imaging characteristics to define DCIS response are lacking and therefore, this remains an important area for future research to refine surgical decision-making following NACT [5].

Thirdly, the authors addressed that the omission of imaging-based response assessment is a missed opportunity as contrast-enhanced MRI (CE-MRI) may play a role in guiding de-escalation to breast-conserving surgery. We agree that the higher mastectomy rates in patients with residual DCIS emphasize the need for predictive markers. While CE-MRI holds promise for assessing DCIS response to NACT, its precise role remains to be defined. The omission of imaging-based response assessment in our study was a limitation, as a result of our retrospective database. Future studies integrating imaging-based predictors may help select patients for breast-conserving surgery.

Lastly, the authors argue that the lack of molecular profiling of DCIS is a notable limitation. We acknowledge that the absence of molecular profiling of DCIS in our study limits conclusions regarding response based on DCIS subtype. However, this is an inherent limitation of our retrospective database. The potential impact of proliferative markers such as Ki67 on DCIS response to NACT, as suggested by the authors, is an intriguing hypothesis that warrants further investigation. While we accounted for other clinicopathological variables, such as DCIS grade and the presence of comedonecrosis and calcifications, the retrospective nature of our study resulted in a high numbers of missing data within these variables. Nevertheless, given the limited number of studies focusing specifically on DCIS response to NACT, we believe our work represents an important step toward addressing this knowledge gap.

In conclusion, while we recognize the limitations inherent in our study, our findings contribute to the evolving understanding of DCIS response to systemic therapy. The notion that extensive DCIS necessitates mastectomy in all cases is increasingly being challenged, and accumulating evidence supports breast-conserving approaches in selected patients. Further research, particularly integrating imaging and molecular profiling, is needed to refine patient selection and optimize surgical decision-making following NACT. We look forward to further research into the response of DCIS to NACT in order to optimize treatment options for patients.

## CRediT authorship contribution statement

**Eva L. Claassens:** Conceptualization, Writing – original draft. **Roxanne A.W. Ploumen:** Writing – review & editing. **Thiemo J.A. van Nijnatten:** Supervision, Writing – review & editing. **Marjolein L. Smidt:** Supervision, Writing – review & editing.

## Sources of funding

This research did not receive any specific grant from funding agencies in the public, commercial, or not-for-profit sectors. None of the authors received support from any organization for the submitted work.

## Declaration of competing interest

Prof. Dr. Marjolein L. Smidt received funding from 10.13039/100031219Nutricia and 10.13039/501100011725Servier Pharmaceuticals for Microbiome research in colorectal cancer, and use of material from Illumin, not related to this study. Dr. Thiemo van Nijnatten received speaker honoraria and institutional grant support from 10.13039/100006775GE Healthcare and 10.13039/100004326Bayer, not related to this study. The other authors have no conflict of interest to declare.
